# Maternal Folic Acid and Dietary Folate Intake in Relation to Sex Ratio at Birth and Sex-Specific Birth Weight in China

**DOI:** 10.3390/nu16183122

**Published:** 2024-09-16

**Authors:** Binyan Zhang, Baibing Mi, Shaonong Dang, Hong Yan

**Affiliations:** 1 School of Public Health, Xi’an Medical College, Xi’an 710021, China; zhangbinyan@stu.xjtu.edu.cn; 2 Department of Epidemiology and Health Statistics, School of Public Health, Health Science Center, Xi’an Jiaotong University, Xi’an 710061, China; xjtu.mi@xjtu.edu.cn (B.M.); yanhonge@xjtu.edu.cn (H.Y.)

**Keywords:** folic acid, dietary folate, sex ratio at birth, sex-specific birth weight

## Abstract

Background: It is well-established that prenatal folic acid supplements can reduce neural tube defects. However, the associations between folic acid supplementation, dietary folate intake, and overall folate intake with sex-specific birth outcomes are not yet fully understood. Objectives: This study aims to investigate the association of periconceptional folic acid supplement, dietary folate, and total folate intake with the sex ratio at birth and sex-specific birth weight. Methods: Data were sourced from a cross-sectional survey conducted between August and December 2013 in Northwest China, involving 7318 infants and their mothers, recruited using a stratified multistage random sampling method. Folic acid supplements (400 μg/d) were ascertained via a retrospective in-person interview. Dietary folate was evaluated using a validated food frequency questionnaire. Birth outcomes, including sex and weight at birth, were obtained from the Medical Certificate of Birth. Generalized linear models were employed to calculate relative risks (RRs) or differences with 95% confidence intervals (CIs). Results: No association or dose–response relationship was observed between folic acid supplement, dietary folate, and total folate intake during periconception and the likelihood of male births. However, women who took folic acid supplements during pre- and post-conception were associated with an increased male birth weight by 52.8 (8.1 to 97.5) g. Additionally, the total folate intake during periconception was associated with birth weight for males (upper vs. lower tertile: β = 38.8, 95%CI: 5.0 to 72.5 g, *p*-trend = 0.024) and females (upper vs. lower tertile: β = 42.4, 95%CI: 6.7 to 78.1; *p*-trend = 0.022). Conclusions: Our findings indicate that periconceptional total folate intake does not correlate with sex ratio at birth but was positively linked to infant birth weights, regardless of gender. These findings offer novel insights into potential benefits of total folate intake, beyond the prevention of neural tube defects, for policymakers and public health.

## 1. Introduction

The sex ratio at birth (SRB), measured as the number of males per 100 female live births, is often considered to be around 105. A systematic SRB estimation across 202 countries from 1950 to 2017 reveals 12 nations with significant SRB imbalances from 1970 to 2017, culminating in a global deficit of 23.1 million female births, especially in China and India [1,2,3]. As high SRB has produced a series of alarming societal consequences, China’s government has made great efforts to promote gender equality; for example, by preventing illegal use of ultrasound technology, providing financial incentives to couples who give birth to girls, or directly promoting cultural changes to move away from patriarchal tradition [4,5]. With the government’s advocacy of gender equality and son preference attitudes gradually changing, the SRB is gradually decreasing, but it still deviates from the biological value 105 [6]. Therefore, it is necessary to further explore the factors that affect the SRB to promote the demographic and social balance of society. SRB may be linked to various factors, such as sociodemographic characteristics, ambient temperature [7], and latitude [8], as well as dietary features. Findings from British women suggest that a higher intakes of nutrients with folate before conception and during early pregnancy are more likely to result in male offspring [9], whereas studies in South American countries and China suggest that the population sex ratio did not change after the folic acid fortification and multivitamin supplementation containing folic acid [10,11]. Also, previous studies do not simultaneously evaluate the association of folic acid supplements, dietary folate, and total folate intake during the periconceptional period with SRB. Further, prenatal folic acid supplements could reduce neural tube defects (NTDs) [12], However, the relationship between folic acid, dietary folate, and other birth outcomes has not been investigated. Therefore, the current study aims to assess the association of periconceptional folic acid supplements, dietary folate, and total folate intake with both SRB and sex-specific birth weight among Chinese populations in order to fill this gap.

## 2. Materials and Methods

### 2.1. Study Design and Population

**Figure 1:**
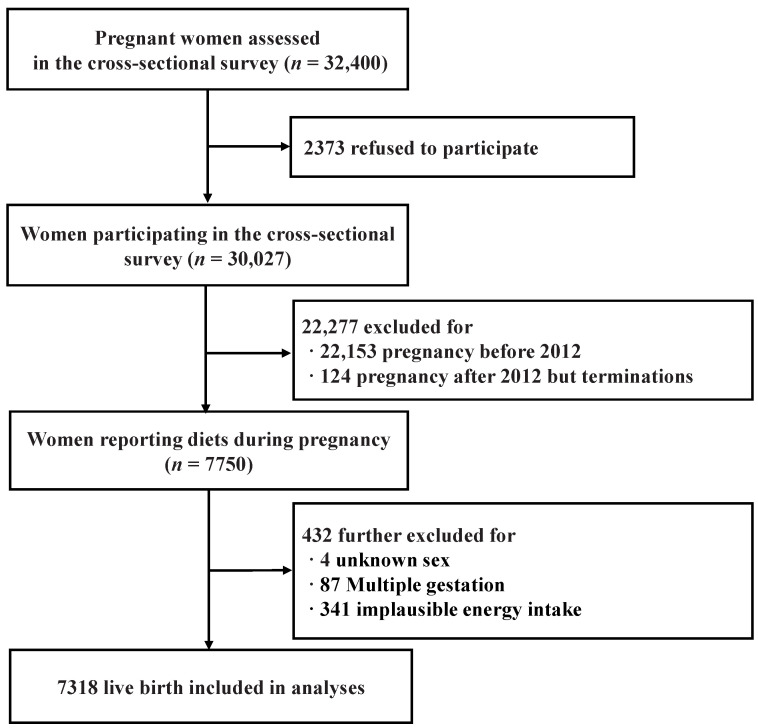
The flow diagram with exclusion criteria.

A cross-sectional epidemiological survey aiming to investigate the risk factors of birth outcome was conducted between August and December 2013 in Shaanxi Province, Northwest China. Infants born during 2010–2013 and their mothers were recruited using a stratified multistage random sampling method; detailed descriptions of the study protocol were available elsewhere [13,14], but briefly, this was performed according to the proportion of the rural–urban distribution and fertility rates in Shaanxi Province, China. Initially, twenty counties and ten districts were randomly selected. Subsequently, six villages from each of six townships in the counties, and six communities from each of three streets in the districts were chosen at random. Finally, thirty individuals were sampled from each village, and sixty from each community. Women of childbearing aged 15–49 were recruited for retrospective interviews to collect data on sociodemographics, reproductive history, and periconceptional folic acid and dietary intake using a family questionnaire. Birth outcome data were sourced from medical birth records. Of the 32,400 pregnant women surveyed, 30,027 (92.7% response rate) completed the questionnaire. Exclusions were made for those with pregnancies before 2012 (*n* = 22,153), or after 2012 with termination (*n* = 124). A total of 7750 women were further interviewed to report dietary intake during the periconceptional period. An additional 432 were excluded due to unknown sex (*n* = 4), multiple gestations (*n* = 87), or implausible energy intake (<500 or >3500 kcal/d, *n* = 341) [15]. The final analysis included 7318 live births, with exclusion criteria detailed in Figure 1.

The study was approved by the Human Research Ethics Committee at Xi’an Jiaotong University Health Science Center (No. 2012008), with written informed consent obtained from all participants.

### 2.2. Ascertainment of Folic Acid Supplementation and Folate Intake

In this survey, folic acid supplements (400 μg/d) during periconception were collected via a retrospective in-person interview through the following stages: pre-conceptional use only (12 weeks before pregnancy), postconceptional use only (1–12 weeks during pregnancy), and both pre- and postconceptional use, respectively. Women with folic acid supplements at any of the stages described above were defined as users across the periconceptional period. Non-users were defined as those who never took folic acid at any of the stages mentioned. Women were also asked to report the brand, duration, and total days of intake of the folic acid supplements. This information was used to calculate the daily mean intake (μg/d) of supplemental folic acid during the periconceptional period, which was further divided into 3 categories: 0, 1–399, and ≥400 μg/d.

The Food Frequency Questionnaire (FFQ) focuses on long-term exposure measurements, resulting in total and general information over a considerable time-period. Considering the slight change in the perinatal dietary pattern [16], information on dietary intake during the periconceptional period (12 weeks before or after pregnancy) was estimated from a semi-quantitative FFQ adapted from an existing FFQ that had been validated for use in pregnant women in rural China [17]. The FFQ included 107 items on common food and beverages, which were organized into 16 groups: (1) seasoning; (2) cereals; (3) tuber products; (4) starch products; (5) soybean products; (6) vegetables; (7) fruits; (8) fish and seafood; (9) dairy products; (10) meat; (11) poultry; (12) eggs; (13) nuts; (14) snacks and sweet desserts; (15) tea and coffee; and (16) fruit juices and soft drinks. For the first group, seasoning, with five food items (vegetable oils, animal oils, salt, sauces, and sugar), the frequency scale of each was determined by an open-ended question, recording as kilograms ingested per month and number of users. For the other 102 items, the women were asked to report the frequency with eight response categories for each item, ranging from ‘almost never’ to ‘two or more times per day’, and each item’s intake quantity was gathered based on the food photographs, which featured three categories of images, large, medium, and small, aiding in portion size estimation, and specified serving sizes described by using natural portions or standard weight and volume measures of the servings commonly consumed in this survey population (e.g., 1 apple, 2 slices of watermelon). Then, the selected frequency category for each food item was converted to a daily intake. The daily intake of dietary folate was calculated using interactive matrix language (IML), by referring to the China Food Composition Table [18,19].

Then, we used the residual method to estimate energy-adjusted values for dietary folate intake to eliminate the effect of total energy, as energy intake was closely related to micronutrient intake [15]. And the dietary folate intake during periconception was classified according to tertiles of daily intake. The total folate intake during periconception equaled the micrograms of food folate plus 1.7 times the micrograms of the folic acid supplement, as synthetic folic acid was more readily bioavailable than natural folates found in foods [20]. Similarly, the total folate intake was classified according to tertiles of daily intake.

### 2.3. Assessment of Birth Outcomes

Information on birth outcomes, including birth sex, birth weight, and gestational age, were abstracted from the Medical Certificate of Birth. The sex of all live births from all gestations ≥28 weeks was considered in this study. The sex at birth of infants was recorded, including any unknown sex. Women who delivered babies of unknown sex were excluded from the final analysis. SRB was defined as the number of males for every 100 females. Birth weight was accurate to 0.1 g.

### 2.4. Assessment of Covariates

Questionnaires were administered through retrospective in-person interviews to collect participant data. Potential confounders were selected based on a literature review [9,21,22,23,24] and univariate analysis results. The final models adjusted for factors such as geographic area (northern, southern, or central Shaanxi), maternal age at delivery (in years), maternal occupation (farmer or other), maternal education (primary school or lower, Junior high school, high school or higher), parity (primiparous or multiparous), household wealth index (poor, medium, rich), smoking (yes or no), pregnancy complications (yes or no), maternal energy (continuous), and gestational age (in weeks). The household wealth index was calculated using principal component analysis with four variables representing the family economic level (monthly income, monthly expenditure, housing condition, and vehicle); a higher household wealth index indicated women with higher economic status, which was categorized as poor, medium, and rich according to tertiles [25]. Maternal smoking was defined as active and passive smoking in the present analysis. Pregnancy complications included gestational hypertension and gestational diabetes.

### 2.5. Statistical Analysis

Continuous variables were presented as mean (SD) or medians with interquartile ranges (IQR) based on data distribution, as dietary folate intake and total folate intake had a positive-skewed distribution and categorical variables as numbers with proportions. Student’s t-test was used for continuous variables, and Pearson’s χ^2^ for categorical variables in univariate analyses. Considering that this survey, using a stratified multistage random sampling method, had a hierarchical structure, the four-level empty models of the county (district)-township (community)-village (street)-individual were fitted and low interclass correlations (all ICC < 0.001) with non-significant within-group variations (all *p*-value > 0.05) were observed at the county, township, and village levels. A generalized linear model at the individual level, therefore, were used to estimate the relative risks (RRs) or difference with 95% confidence intervals (CIs) for associations of periconceptional folic acid supplement and folate intake with male delivery and sex-specific birth weight after adjusting for confounding factors, separately. Dose–response relationships with *p*-trend were obtained using the median value of each group folic acid supplement or folate intake as a continuous variable in each model after controlling for confounding factors. In addition, the associations of different timings of periconceptional folic acid supplement with the adjusted RR for sex ratio and sex-specific birth weight were explored.

Regarding the missing data, we conducted multiple imputation via a fully conditional specification approach with ten burn-in iterations for all participants, generating five multiply imputed datasets, which was sufficient to provide a robust estimate of variance [26]. Then, the results of the analyses from imputed datasets were further combined and generated validly comprehensive statistical inferences. The discriminant function method for classification covariates and the regression method for continuous covariates were used to impute missing values. Furthermore, the distribution equalization of the observed and the imputed data for each variable was compared using the Kolmogorov–Smirnov test to determine missingness at random or not, and the distributions of the observed and the imputed data were also examined visually [27]. Finally, we ran our main analyses in the imputed sample (*n* = 7318).

Statistical analyses were conducted with SAS software (version 9.4; SAS Institute Inc., Cary, NC, USA). A two-tailed *p*-value of less than 0.05 was considered to indicate statistical significance.

## 3. Results

### 3.1. Participant Characteristics

Table 1 presents the characteristics of pregnant women and infants based on periconceptional folic acid supplement use. No significant differences were found in maternal age, pregnancy complications, gestational age, infant sex, or male birth weight between the supplemented and non-supplemented groups. Compared to non-users, folic acid users were more frequently from central and southern Shaanxi, more primiparous, non-farmers, had higher levels of education and household wealth index, were less likely to smoke, and exhibited higher energy intake, as well as heavier female birth weights (all *p*-value < 0.05). Table 2 shows that primiparous women were more likely to give birth to female infants than multiparous ones (*p*-value = 0.018), and male infants had a shorter gestational age and higher mean birth weight than females (*p*-value < 0.001). However, no significant differences were observed in geographic area, maternal age, education, occupation, or household wealth index, smoking, pregnancy complications, or maternal energy intake between the male and female infant groups (*p*-value > 0.05).

### 3.2. Periconceptional Folic Acid Supplements and Sex Ratio at Birth and Sex-Specific Birth Weight

Among the 7318 pregnant women, 5165 (70.6%) took folic acid during the periconceptional period. The supplementation rates were 5.7% (*n* = 414), 50.7% (*n* = 3707), and 14.3% (*n* = 1044) for the preconceptional, postconceptional, and both periods, respectively. Women taking 1–399 μg/d and ≥400.0 μg/d of folic acid constituted 65.7% and 4.9% of the sample, separately. Folic acid supplementation was not significantly associated with the relative risk of male birth, irrespective of the timing of supplementation. Similarly, no association was observed between daily folic acid dosage and SRB (*p*-trend = 0.510). However, women who supplemented during both pre- and postconceptional periods were associated with increased birth weights by 46.2 g (95%CI: 13.3 to 79.1) for all infants and 52.8 g (95%CI: 8.1 to 97.5) for male infants. In addition, we observed a potential dose–response relation between folic acid supplement and infant birth weight (*p*-trend = 0.042), Table 3.

### 3.3. Periconceptional Folate Intake and Sex Ratio at Birth and Sex-Specific Birth Weight

The median intakes of dietary folate were 236.9 (IQR 123.3), 157.8 (IQR 61.0), 236.9 (IQR 35.8), and 347.2 (IQR 118.0) μg/d across the periconceptional period and each tertile, separately. Likewise, the median total folate intakes were 545.0 (IQR 571.6), 223.8 (IQR 102.9), 545.1 (IQR 205.4), and 907.9 (IQR 137.8) μg/d among corresponding groups. A significant dose–response relationship was observed between dietary folate and both overall (*p*-trend = 0.010) and male birth weight (*p*-trend = 0.046). Being in the highest tertile of total folate intake was related to a 39.7 g increment in birth weight compared to those in the lowest tertile (95%CI: 15.0 to 64.4; *p*-trend = 0.002). Also, dose–response relationships were found between total folate intake and male (*p*-_trend_ = 0.024) and female birth weight (*p*-trend = 0.022); see Table 4.

## 4. Discussion

The present study suggested that periconceptional folic acid supplement was not associated with SRB, whether taken before or after conception or during both periods. Even though we considered the daily dose of folic acid supplement intake, there was no association between folic acid supplement and SRB. Moreover, folic acid supplement, dietary folate, and total folate intake during periconception were not linked to SRB. On the other hand, we found that supplemental folic acid, dietary folate, and total folate were positively associated with birth weight during the periconceptional period.

We observed that the SRB did not change when women took folic acid during the periconceptional period, which was consistent with previous studies [10,11]. Also, we found that there was no relationship of folic acid supplement, dietary folate, and total folate intake with SRB, which further provided evidence that neither folic acid supplement nor dietary folate was associated with SRB.

In the most stable human population, the biologically normal SRB ranged from 102 to 107 males per 100 females. However, the SRB in the present study was 116; this figure deviated from its biologically common value. Such deviations may be attributed to maternal demographic factors and environmental exposures, including but not limited to famine [28], toxins [29,30], and the aftermath of earthquakes [31]. Furthermore, a substantial hospital-based study conducted in Nepal indicated that sex-selective abortion was a predominant factor contributing to an imbalanced SRB [32].

While the literature indicated that the sex ratio at conception was unbiased [33], emerging data from Britain have shown fetal sex associated with maternal diet at conception. Furthermore, an evolutionary theory regarding sex ratio variation proposed that parents in optimal condition were more likely to conceive male offspring [34]. In fact, the genetic dynamics and demographics of the sex ratio from pregnancy to birth remain an area of ongoing investigation. It is well-established that the sex of offspring is subject to the influences of natural selection. Recent research had reported a positive association between maternal preconception vitamin D concentrations and the live birth of male infants [35]. Additionally, a study has indicated that both dietary and in vitro supplementation with omega-6 polyunsaturated fatty acids could skew the sex ratio towards the male side [36]. As we know, the first trimester of pregnancy is associated with inflammation, which is a necessary process for blastocyst implantation [37]. Vitamin D, omega-6 polyunsaturated fatty acids, and folate might all have anti-inflammatory properties [38], which could potentially mitigate maternal inflammation. This modulation of the inflammatory environment might be crucial for the successful implantation or survival of male fetuses in utero, thereby influencing the observed sex ratio. The periconceptional period is a critical period for the differentiation and formation of fetal sex, and coincides with the stage of folic acid supplement to prevent NTDs. In the present study, we provided new evidence that women’s intake of folic acid or/and folate is not linked to SRB. In spite of the biological plausibility, the possibility of our findings might be influenced by induced abortions and artificial intervention pregnancy terminations, so we must be cautious in the interpretation of the findings.

Moreover, our research suggests that total folate intake was positively associated with birth weight, indicating that folate might provide a benefit for infants’ birth weight gain in addition to preventing NTDs. This finding aligned with those of a preceding study [39]. Folate is vital for fetal growth and a critical component in one-carbon metabolism. Whereas one-carbon metabolism ultimately provides methyl for biochemical reactions, the establishment of epigenetic patterns in early pregnancy is susceptible to folate intake. The California Childhood Leukemia Study had identified associations between periconceptional folate consumption and offspring DNA methylation that varied by dietary source, suggesting that the source of folate might be associated with early DNA methylation patterns [40]. Therefore, folate deficiency during periconception might lead to the impaired metabolism of one-carbon and homocysteine accumulation, triggering oxidative stress and placental dysfunction, which could culminate in low birth weight.

The strength of the present study was a larger population-based study with a stratified multistage random sampling method in Shaanxi Province, Northwest China, and the sample obtained was relatively representative. Another important strength was that we collected detailed information on both folic acid supplement and dietary folate intake. However, several limitations in the present study should be acknowledged. First, no causality could be determined due to the cross-sectional design; although quality control measures were taken to help participants recall accurately before the formal field investigation, including the compilation of objective standard questionnaires and in-person interviews with graphical information about the weight of each food, recall bias might still be unavoidable. Second, despite controlling for confounding factors, we cannot rule out the possibility of residual confusion from unknown confounders and the absence of data on maternal height and weight gain. Third, the length of the FFQ might increase the burden on the women, thereby impairing the respondents’ cooperation and increasing the risk of response bias and overestimation of intake. Therefore, considering this limitation, the FFQ was managed by a strictly trained interviewer to ensure the accurate completion of the answers [41]. Accordingly, previous studies indicated that foods with a food list of more than 100 items performed better than those with a shorter length of 100 foods, so the burden on respondents was not a decisive factor for FFQ [42].

## 5. Conclusions

Our research indicates no association between periconceptional total folate intake and the sex ratio at birth, yet identifies a significant positive association with infant birth weight, irrespective of gender. These findings offer novel insights into the safety and potential benefits of folic acid and folate intake, beyond the prevention of NTDs, for policymakers and public health concerns. A future well-designed study could delve into the underlying mechanisms linking folate intake to birth weight and assess the impact of folate-related dietary patterns on birth weight to inform public health practices aimed at enhancing maternal and infant well-being.

## Figures and Tables

**Table 1:** Characteristics of women and infants according to the status of periconceptional folic acid use in Shaanxi Province, Northwest China.

Characteristics	No Folic Acid (*n* = 2153)		Folic Acid (*n* = 5165)		*p*-Value
	*n*	%	*n*	%	
Geographic area					<0.001
Northern Shaanxi	598	27.8	743	14.4	
Central Shaanxi	973	45.2	2854	55.3	
Southern Shaanxi	582	27.0	1567	30.3	
Maternal age at delivery, y, mean (SD)	27.5 (5.2)		27.4 (4.3)		0.392
Maternal parity					<0.001
Primiparous	1151	53.5	3325	64.4	
Multiparous	1002	46.5	1840	35.6	
Maternal education					<0.001
Primary school or below	313	14.6	354	6.9	
Junior high school	1291	60.1	2628	51.1	
Senior high school or above	544	25.3	2166	42.1	
Farmer	1666	78.1	3589	70.0	<0.001
Household wealth index					<0.001
Poor	860	39.9	1589	30.8	
Medium	694	32.2	1717	33.2	
Rich	599	27.8	1859	36.0	
Smoking	296	13.8	419	8.1	<0.001
Pregnancy complications	31	1.4	84	1.6	0.559
Energy, kcal/d, mean (SD)	1555.9 (542.8)		1613.39 (562.7)		<0.001
Gestational age, wk, mean (SD)	39.6 (1.3)		39.6 (1.3)		0.125
Sex at birth					0.227
Male	1180	54.8	2751	53.3	
Female	973	45.2	2414	46.7	
Birthweight, g, mean (SD)					
Male	3298.1 (466.3)		3328.8 (437.0)		0.056
Female	3181.7 (462.7)		3217.2 (428.6)		0.041

Values for some characteristics may not be equal to total numbers of folic-acid or non-folic-acid groups because of missing values. The number of missing values was 60 cases for maternal age at delivery, 22 cases for maternal education, and 54 cases for maternal occupation. Pregnancy complications include gestational hypertension and gestational diabetes. d = day, wk = weeks, y = years.

**Table 2:** Characteristics of women and infants according to infant sex at birth in Shaanxi Province, Northwest China.

Characteristics	Female (*n* = 3387)		Male (*n* = 3931)		*p*-Value
	*n*	%	*n*	%	
Geographic area					0.700
Northern Shaanxi	607	17.9	734	18.7	
Central Shaanxi	1776	52.5	2051	52.2	
Southern Shaanxi	1003	29.6	1146	29.2	
Maternal age at delivery, y, mean (SD)	27.5 (4.6)		27.3 (4.6)		0.296
Maternal parity					0.018
Primiparous	2121	62.6	2355	59.9	
Multiparous	1266	37.4	1576	40.1	
Maternal education					0.768
Primary school or below	306	9.1	361	9.2	
Junior high school	1803	53.4	2116	54.0	
Senior high school or above	1270	37.6	1440	36.8	
Farmer	2438	72.5	2817	72.2	0.788
Household wealth index					0.203
Poor	1126	33.2	1323	33.7	
Medium	1150	34.0	1261	32.1	
Rich	1111	32.8	1347	34.3	
Smoking	317	9.4	398	10.1	0.272
Pregnancy complications	59	1.7	56	1.4	0.276
Energy, kcal/d, mean (SD)	1590.4 (561.6)		1601.5 (554.0)		0.058
Gestational age, wk, mean (SD)	39.6 (1.3)		39.5 (1.3)		<0.001
Birthweight, g, mean (SD)	3207.1 (438.8)		3319.6 (446.1)		<0.001

Values for some characteristics may not be equal to total numbers of female or male groups because of missing values. The number of missing values was 60 cases for maternal age at delivery, 22 cases for maternal education, and 54 cases for maternal occupation. Pregnancy complications include gestational hypertension and gestational diabetes.

**Table 3:** The association of periconceptional folic acid supplement with adjusted relative risks for sex at birth or difference in birthweight in Shaanxi Province, Northwest China. (*n* = 7318).

	Male *n* (%)	Female *n* (%)	SRB	SRB	Birthweight (g)	Male Birthweight (g)	Female Birthweight (g)
				Adjusted RR (95%CI)	Adjusted β (95%CI)	Adjusted β (95%CI)	Adjusted β (95%CI)
Non-users	1180 (54.8)	973 (45.2)	121	Ref.	Ref.	Ref.	Ref.
Users	2751 (53.3)	2414 (46.7)	114				
Preconception	216 (52.2)	198 (47.8)	109	0.96 (0.87 to 1.07)	29.0 (−17.1 to 75.0)	47.8 (−15.4 to 111.1)	10.7 (−55.3 to 76.6)
Postconception	1978 (53.4)	1729 (46.6)	114	0.98 (0.93 to 1.03)	19.2 (−4.1 to 42.5)	23.8 (−7.9 to 55.5)	16.7 (−17.1 to 50.5)
Pre-and postconception	557 (53.4)	487 (46.7)	114	0.99 (0.92 to 1.06)	46.2 (13.3 to 79.1)	52.8 (8.1 to 97.5)	37.9 (−9.7 to 85.6)
Folic acid, μg/d, median (IQR)							
0.0	1180 (54.8)	973 (45.2)	121	Ref.	Ref.	Ref.	Ref.
1.0–399.0, 144.4 (100.0)	2552 (53.1)	2253 (46.9)	113	0.99 (0.94 to 1.04)	15.6 (−7.7 to 38.9)	18.6 (−13.2 to 50.3)	13.4 (−20.3 to 47.1)
≥400.0, 400.0 (0.0)	199 (55.3)	161 (44.7)	124	0.98 (0.93 to 1.04)	26.6 (0.8 to 52.5)	27.3 (−8.0 to 62.7)	28.0 (−9.2 to 65.3)
*p*-trend				0.510	0.042	0.123	0.141

SRB: number of males for every 100 females; RR: Relative risk; Ref., reference; IQR, interquartile range. RRs for sex ratio at birth were estimated using the generalized linear model with binomial distribution and log-link function after adjusting for confounding factors, including geographic area, maternal age at delivery, maternal parity, maternal education, occupation, household wealth index, and maternal energy intake. β was estimated using generalized linear model with normal distribution and identity-link function after adjusting for confounding factors, including geographic area, maternal age at delivery, maternal parity, maternal education, occupation, household wealth index, maternal energy intake, smoking, pregnancy complications, and gestational age. *p*-trend was obtained by using the median value of each group’s folic acid supplement as a continuous variable.

**Table 4:** The association of periconceptional dietary folate and total folate intake with adjusted relative risks for sex at birth or difference in birthweight in Shaanxi Province, Northwest China (*n* = 7318).

	Male *n* (%)	Female *n* (%)	SRB	SRB	Birthweight (g)	Male Birthweight (g)	Female Birthweight (g)
				Adjusted RR (95%CI)	Adjusted β (95%CI)	Adjusted β (95%CI)	Adjusted β (95%CI)
Dietary folate intake, μg/d, median (IQR)							
Tertile 1, 157.8 (61.0)	1320 (54.1)	1119 (45.9)	118	Ref.	Ref.	Ref.	Ref.
Tertile 2, 236.9 (35.8)	1316 (53.9)	1124 (46.1)	117	1.00 (0.95 to 1.06)	−11.1 (−36.0 to 13.8)	−10.8 (−44.7 to 23.0)	−10.8 (−46.9 to 25.3)
Tertile 3, 347.2 (118.0)	1295 (53.1)	1144 (46.9)	113	0.99 (0.94 to 1.04)	29.0 (4.5 to 53.5)	30.9 (−2.5 to 64.2)	28.7 (−6.7 to 64.1)
*p*-trend				0.704	0.010	0.046	0.077
Total folate intake, μg/d, median (IQR)							
Tertile 1, 223.8 (102.9)	1330 (54.5)	1110 (45.5)	120	Ref.	Ref.	Ref.	Ref.
Tertile 2, 545.1 (205.4)	1305 (53.5)	1134 (46.5)	115	0.99 (0.94 to 1.04)	23.2 (−1.2 to 47.6)	19.0 (−14.2 to 52.2)	30.0 (−5.3 to 65.3)
Tertile 3, 907.9 (137.8)	1296 (53.1)	1143 (46.9)	113	0.98 (0.93 to 1.04)	39.7 (15.0 to 64.4)	38.8 (5.0 to 72.5)	42.4 (6.7 to 78.1)
*p*-trend				0.534	0.002	0.024	0.022

SRB: number of males for every 100 females; RR: Relative risk; Ref., reference; IQR, interquartile range. RRs for sex ratio at birth were estimated using the generalized linear model with binomial distribution and log-link function after adjusting for confounding factors, including geographic area, maternal age at delivery, maternal parity, maternal education, occupation, household wealth index, and maternal energy intake. β was estimated using the generalized linear model with normal distribution and identity-link function after adjusting for confounding factors, including geographic area, maternal age at delivery, maternal parity, maternal education, occupation, household wealth index, maternal energy intake, smoking, pregnancy complications, and gestational age. *p*-trend was obtained by using the median value of each group’s folate intake as a continuous variable.

## Data Availability

The data referenced in this article are available upon reasonable request to the corresponding author, Shaonong Dang (tjdshn@xjtu.edu.cn). Data is not available publicly due to an ongoing study.
